# The internal interaction in RBBP5 regulates assembly and activity of MLL1 methyltransferase complex

**DOI:** 10.1093/nar/gkz819

**Published:** 2019-09-23

**Authors:** Jianming Han, Tingting Li, Yanjing Li, Muchun Li, Xiaoman Wang, Chao Peng, Chen Su, Na Li, Yiwen Li, Ying Xu, Yong Chen

**Affiliations:** 1 State Key Laboratory of Molecular Biology, National Center for Protein Science Shanghai, CAS Center for Excellence in Molecular Cell Science, Shanghai Institute of Biochemistry and Cell Biology, Chinese Academy of Sciences; University of Chinese Academy of Sciences, 333 Haike Road, Shanghai 201210, China; 2 School of Life Science and Technology, Shanghai Tech University, 100 Haike Road, Shanghai 201210, China; 3 National Facility for Protein Science in Shanghai, Zhangjiang Lab, Shanghai 201210, China; 4 Shanghai Science Research Center, Chinese Academy of Sciences, Shanghai 201204, China

## Abstract

The Mixed Lineage Leukemia protein 1 (MLL1) plays an essential role in the maintenance of the histone H3 lysine 4 (H3K4) methylation status for gene expression during differentiation and development. The methyltransferase activity of MLL1 is regulated by three conserved core subunits, WDR5, RBBP5 and ASH2L. Here, we determined the structure of human RBBP5 and demonstrated its role in the assembly and regulation of the MLL1 complex. We identified an internal interaction between the WD40 propeller and the C-terminal distal region in RBBP5, which assisted the maintenance of the compact conformation of the MLL1 complex. We also discovered a vertebrate-specific motif in the C-terminal distal region of RBBP5 that contributed to nucleosome recognition and methylation of nucleosomes by the MLL1 complex. Our results provide new insights into functional conservation and evolutionary plasticity of the scaffold protein RBBP5 in the regulation of KMT2-family methyltransferase complexes.

## INTRODUCTION

The methylation of Histone H3 Lysine 4 (H3K4) is crucial to the epigenetic regulation of gene transcription (1,2). The dimethylation and trimethylation of H3K4 mainly occur at the promoters and coding regions of actively transcribed genes, while H3K4 monomethylation is primarily enriched at the enhancers (3,4). The methylation of H3K4 is predominantly mediated by the KMT2-family histone methyltransferases (5,6). Set1 is the only KMT2-family protein discovered in *Saccharomyces cerevisiae* (7,8). At least six Set1 homologs have been identified in mammalian cells, including SET1A, SET1B, and four Mixed Lineage Leukemia proteins (MLL1, MLL2, MLL3, and MLL4) (5,6). Of these, MLL1, the founding member of KMT2-family methyltransferase, has drawn the most attention because chromosome translocations of MLL1 lead to acute lymphoid and myeloid leukemia (9).

Although MLL1 and other KMT2-family proteins contain a conserved C-terminal catalytic SET [SU(VAR)3–9, E(Z) and TRX] domain, their intrinsic histone lysine methyltransferase (HKMT) activities remain low (10). Three regulatory proteins, WDR5, ASH2L and RBBP5, can bind to MLL1 and stimulate the HKMT activity of MLL1 (10,11). WDR5, ASH2L and RBBP5 are conserved from yeast to human (5,12). Their yeast homologs Swd3, Bre2, and Swd1 are also irreplaceable for the assembly and activity regulation of the yeast Set1-containing complex, COMPASS (COMplex of Proteins Associated with Set1) (6).

Recent studies on the structure of KMT2-family complexes have shed light on how these regulatory proteins bind and activate KMT2-family proteins (13–15). The splA and ryanodine receptor (SPRY) domain of ASH2L (ASH2L_SPRY_, residues 286–505) forms a heterodimer with a short fragment of RBBP5 (RBBP5_AS-ABM_, residues 330–360) to stimulate the HKMT activity of MLL-family methyltransferases (15). The crystal structure of the minimized MLL3_SET_-RBBP5_AS-ABM_–ASH2L_SPRY_ complex revealed that RBBP5_AS-ABM_–ASH2L_SPRY_ constrained the flexibility of the SET-I motif of MLL_SET_, thereby facilitating substrate binding and catalytic activation (15). Furthermore, the structures of yeast COMPASS complexes revealed the assembly and regulation mechanisms of an active holoenzyme (13,14). The yeast COMPASS complex adopts a Y-shaped structure in which Swd1 (an RBBP5 ortholog) and Swd3 (a WDR5 ortholog) form the two arms of the Y-shape. Set1 sits at the central fork and Bre2 (an ASH2L ortholog) is located at the bottom of the Y-shape (13,14). Low-resolution cryo electron micoscropy analysis suggested that the MLL1 core complex may adopt a similar architecture as the yeast COMPASS complex (16). However, the detailed structural models of different human KMT2-family complexes remain to be determined.

**Figure 1.**
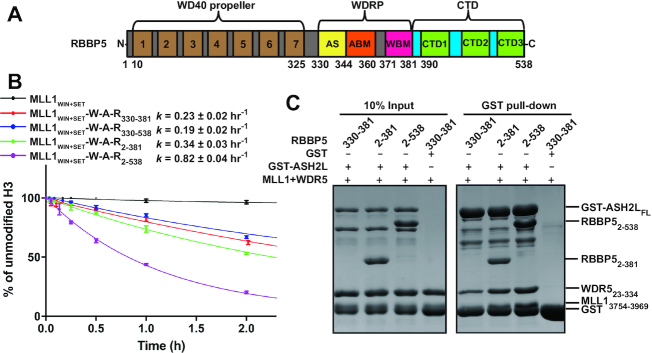
The WD40 and CTD regions of RBBP5 are important for activity regulation of the MLL1 complex. (**A**) Domain organization of human RBBP5. The seven repeats of the WD40 propeller are indicated. WDRP, WD40 Repeat Proximity domain; AS, Activation Segment; ABM, ASH2L-Binding Motif; WBM, WDR5-Binding Motif; CTD, C-Terminal Distal domain. (**B**) Comparison of the overall rates of the methylation reactions on H3 peptides catalyzed by the MLL1 complex assembled with different RBBP5 fragments. Each data point was shown as mean ± s.d. from triplicate measurements. The reported rate constants and fitting errors were derived by exponential fitting of the decrease in the relative intensities of the unmodified H3 peptide peaks in MALDI–TOF mass spectra. (**C**) GST pull-down assays revealed the stabilities of the MLL1 complexes. GST-ASH2L_FL_ was incubated with MLL1_3754–3969_, WDR5_23–334_, and different RBBP5 constructs. Bound proteins were eluted and separated by SDS-PAGE. MLL1_3754–3969_ ran at a similar position as GST as indicated.

Among KMT2-associated regulators, RBBP5 and its yeast ortholog Swd1 play a pivotal role in the assembly and activity regulation of KMT2-family complexes (13–15). Both RBBP5 and Swd1 contain a WD40 propeller domain followed by a long C-terminal tail (17). The C-terminal tail can be further divided into two regions, a WD40 repeat proximal (WDRP) region and a C-terminal distal (CTD) region (Figure 1A) (14). The conserved WDRP region of RBBP5/Swd1 contains three adjacent short motifs that mediate interactions with KMT2_SET_, ASH2L/Bre2, and WDR5/Swd3, respectively (14,15). Although RBBP5 orthologs are highly similar in sequence, they exhibit variations that may relate to distinct roles of RBBP5 in different species. First, the WD40 propeller of human RBBP5 features a ring structure along the axis arising from arginine side-chains (hereinafter referred to as arginine-ring), which is involved in DNA/RNA-binding. This unique feature is absent in yeast Swd1 (17). Second, the C-terminal distal region of RBBP5 is highly variable in length in different species, from 25 aa (residues 403–427) in *Saccharomyces cerevisiae* to 158 aa (residues 381–538) in *Homo sapiens*. *Kluyveromyces lactis* COMPASS structure shows that the short CTD of Swd1 (residues 422–439) extending from the Swd3-binding motif is sandwiched between Swd3_WD40_ and Swd1_WD40_ to further secure Swd3 binding (14). Removal of the CTD_422–439_ from Swd1 completely abolished the binding of Swd3 (14). However, in human RBBP5, the CTD region (residues 381–538) is dispensable for the binding of RBBP5 to WDR5 (18). Therefore, the conformation and function of the extended CTD region in human RBBP5 still require further characterization.

Although RBBP5 and its yeast ortholog Swd1 have been extensively studied, there are still controversies regarding its structural feature and functional roles in the regulation of KMT2-family complex. In our work, we elucidated an internal interaction between the WD40 propeller and the CTD region in human RBBP5 by structural analysis and biochemical assays. This internal interaction promotes a compact conformation of the MLL1 complex and fine-tunes the activity of the MLL1 complex. We also uncovered a novel DNA-binding motif in the CTD region that is involved in nucleosome binding and methylation of nucleosomes mediated by the MLL1 complex.

## MATERIALS AND METHODS

### Protein expression and purification

MLL1_WIN-SET_ (MLL1_3754–3969_) containing both WIN motif and SET domain, full-length RBBP5, full-length ASH2L, and WDR5Δ22 (WDR5_23–334_) were purified as previously described (15). RBBP5 WD40-containing constructs and CTD-containing fragments were expressed as His-Sumo-fusion proteins in Rosetta cells. The proteins were purified by Ni-NTA affinity column, and on-beads digestion using Ulp1 protease. The eluted proteins were further purified by size-exclusion chromatography (SEC) on a Hiload Superdex 75 or 200 column. Purified proteins were concentrated, snap-frozen in liquid nitrogen, and stored at −80°C. Se-Met derivative protein of RBBP5_10–325_ was expressed by growing cells in M9 minimal media and purified by using the same protocol as the native protein. All mutations were introduced by PCR-based site-directed mutagenesis, and mutant proteins were purified by using the same protocol as described above.

### Crystallization, data collection and structural determination

After extensive screening, we obtained the complex crystal from the following constructs: WD40 propeller with residues 10–325 (RBBP5_10–325_) and C-terminal distal region from 390 to 480 with the 422–443 loop deletion (RBBP5_390–480d_). The RBBP5_10–325_-RBBP5_390–480d_ complex was obtained by mixing two separately purified proteins at 1:2 molar ratio, followed by size-exclusion chromatography on a Hiload Superdex 200 column in the buffer containing 150 mM NaCl, 25 mM Tris–HCl, pH 8.0 and 5 mM DTT. Native or Se-Met RBBP5 complex was crystallized in 0.1 M HEPES-Na, pH 7.5, 2% (w/v) PEG 400, 2.0 M ammonium sulfate at 16°C by vapor diffusion method. A 1.8 Å selenomethionine-SAD (single-wavelength anomalous dispersion) dataset was collected at the Se peak wavelength at beamline BL19U1 of National Facility for Protein Science Shanghai (NFPS) in Shanghai Synchrotron Radiation Facility (SSRF). The dataset was processed by HKL3000 (19). The crystal belongs to the P1 space group, and there are two complexes in one asymmetric unit. AutoSHARP was used for selenium site search, solvent flattening, and automatic model building (20). Eight selenium atoms were located and refined, and an initial model was automatically built with good quality using ARP/WARP (21). Crystallography refinement was then carried out in PHENIX (22) together with manual model building in COOT (23).

### GST-pull down assays

GST-ASH2L_FL_, WDR5_23–334_, MLL1_3754–3969_, and RBBP5 proteins were incubated with 10 μl glutathione Sepharose 4B beads (GE Healthcare, USA) in 100 μl binding buffer containing 150 mM NaCl, 25 mM Tris–HCl, pH 8.0, 1 mM PMSF, 10 μM ZnSO4 and 2 mM DTT. After 2 h incubation at 4°C, the resin was washed three times with a total volume of 1.2 ml binding buffer. The bound proteins were then eluted by 25 μl elution buffer (150 mM NaCl, 25 mM Tris–HCl, pH 8.0, 4 mM DTT, 20 mM reduced GSH), and analyzed by SDS-PAGE on 8–12% Tris-glycine gel.

### Isothermal titration calorimetry

The equilibrium dissociation constants between RBBP5_2–333_ and different C-terminal constructs of RBBP5 were determined by the MicroCal iTC200 calorimeter (Malvern Panalytical Ltd, UK). Different C-terminal constructs of RBBP5 (1 mM) were titrated into 0.1 mM RBBP5_2–333_ in the assay buffer of 300 mM NaCl, 25 mM Tris–HCl, pH 8.0, unless stated. To dissect the effect of ion-strength on the interaction between RBBP5_2–333_ and RBBP5_381–538_, three different salt concentrations were tested (150 mM NaCl, 300 mM NaCl and 800 mM NaCl) in the 25 mM Tris–HCl, pH 8.0 buffer. To evaluate the effects of mutations in the CTD region of RBBP5, RBBP5_390–480d_ or its mutants (1 mM) was injected into a sample cell containing 0.1 mM RBBP5_2–333_ in the assay buffer (300 mM NaCl, 25 mM Tris–HCl, pH 8.0). The dissociation constants between different RBBP5 constructs and WDR5 (WDR5_23–334_) were determined in the assay buffer of 150 mM NaCl, 25 mM Tris–HCl, pH 8.0. A total of 20 injections were carried out at 20°C with a reference power of 5 μcal/s. Binding data were plotted and analyzed in Origin 7 (OriginLab, USA). The ITC measurements for each interaction pair were repeated twice. The dissociation constants (*K*_d_) and the fitting errors are derived from one representative ITC curve by data fitting using one-site binding model.

### Fluorescence polarization assay

RBBP5 proteins were serially diluted into the buffer (20 mM HEPES, pH 7.4, 150 mM NaCl, 0.1% BSA) from 400 to 0.2 μM in 12 reactions tubes with a final volume of 30 μl. An equal volume of FAM-labeled 167-bp dsDNA was added to each tube to reach a final concentration of 0.1 μM DNA. The reaction mixture was incubated at 25°C for 30 min in the dark. The fluorescence anisotropy of each tube was measured using Synergy Neo Multi-Mode Reader (BioTek, USA). The *K*_d_ values and the fitting errors are calculated with Prism 6.0 software (GraphPad, USA) by using the sigmoidal dose-response model.

### Biological Small-Angle X-ray Scattering

Small-angle X-ray scattering (SAXS) experiments were performed at beamline BL19U2 of National Facility for Protein Science Shanghai (NFPS) in Shanghai Synchrotron Radiation Facility (SSRF) (24). The wavelength of X-ray is 0.918 Å. Scattered X-ray intensities were collected using a Pilatus 1M detector (DECTRIS Ltd, Switzerland). The sample-to-detector distance was set such that the detecting range of momentum transfer [*q* = 4π sinθ/λ, where 2θ is the scattering angle] of SAXS experiments was 0.008–0.47 Å^−1^. A flow cell made of a cylindrical quartz capillary with a diameter of 1.5 mm and a wall of 10 μm was used. The MLL1 complexes were obtained by mixing MLL1_3754–3969_, WDR5_23–334_, ASH2L_FL_ and RBBP5 at the molar ratio of 2:1:1:1, followed by the purification on the Superdex 200 increase (10/300 GL) column. Then they were diluted to three different concentrations (0.5, 1 and 2 mg/ml) in the buffer of 300 mM NaCl, 25 mM Tris, pH 8.0, 4% glycerol and 1 mM TCEP. SAXS data were collected as 20 × 1-s exposures and scattering profiles for the 20 passes were compared at 10°C using 60 μl sample. The 2D scattering images were converted to 1D SAXS curves through azimuthally averaging after solid angle correction and then normalizing with the intensity of the transmitted X-ray beam, using the software package BioXTAS RAW (25). Background scattering was subtracted using PRIMUS in ATSAS software package (26). Linear Guinier plots in the Guinier region (q*R_g_ < 1.3) were confirmed in all experimental groups. Pair distance distribution functions of the particles P(r) and the maximum sizes *D*_max_ were computed using GNOM (27). The *ab initio* shapes were determined using DAMMIF (28) with 15 DAMMIF runs for each experimental group and DAMAVER (29) was used to analyze the normalized spatial discrepancy (NSD) between the 15 models. The models with an NSD greater than the average NSD ± 2 standard deviation were excluded, and the remaining aligned models were averaged to get the representative molecular envelope.

### Cross-linking mass spectrometry

The MLL1 complex was obtained by mixing MLL1_3754–3969_, WDR5_23–334_, ASH2L_FL_ and RBBP5_FL_ at the molar ratio of 2:1:1:1, followed by the purification on the Superdex 200 increase (10/300 GL) column. The purified complex was diluted to 5 μM in the buffer of 150 mM NaCl, 25 mM HEPES, pH 7.0 with a final volume of 20 μl. EDC (1-ethyl-3-[3-dimethylaminopropyl] carbodiimide hydrochloride) was first mixed with MLL1 complex at a final concentration of 1 mM. After incubation at room temperature for 1 h, the reaction was quenched by addition of 20 mM 2-mercaptoethanol. Then Sulfo-NHS (*N*-hydroxysulfosuccinimide) was added to the reaction mixture at a final concentration of 2 mM, followed by incubation at room temperature for 2 h. Finally, the reaction was quenched by adding 20 mM Tris (pH 8.0). The reaction products were separated by SDS-PAGE on 8–10% Tris-glycine gels and visualized by staining with Coomassie blue.

The gel band of cross-linked MLL1 complex was excised and cut into small pieces. The gel sample was then destained with methanol/H_2_O, followed by TCEP reduction (10 mM), iodoacetamide alkylation (55 mM) and trypsin digestion. Trypsin digestion was performed with sequencing-grade modified trypsin (Promega, USA) at 37°C overnight. The tryptic digested peptides were extracted and desalted with monoSpin C18 columns (GL Science, Japan), followed by separation in a Proxeon EASY-nLC liquid chromatography system (ThermoFisher Scientific, USA) by applying a step-wise gradient of 0–80% acetonitrile in 0.1% formic acid. Peptides eluted from the LC column were directly electrosprayed into the mass spectrometer with a distal 2 kV spray voltage. Tandem mass spectrometry (MS/MS) analysis was performed on a Q-Exactive instrument (ThermoFisher Scientific, USA) in a 120-minute gradient. Cross-linked peptides were identified and evaluated using pLink2 software (30).

### MALDI-TOF-based methyltransferase assay

The MLL1 complex was prepared by mixing MLL1_3754–3969_, WDR5_23–334_, ASH2L_FL_ and RBBP5 at the molar ratio of 1:1:1:1 without further purification. For a 50 μl reaction, 250 μM SAM, 10 μM histone peptide (ARTKQTARY) and 1 μM MLL1 complex were mixed in the buffer containing 20 mM HEPES (pH7.8), 10 mM NaCl, and 5 mM DTT and incubated at 25°C. At different time points, 4 μl reaction mixture was taken out and treated with 0.5% trifluoroacetic acid to terminate the reaction. Then, 1 μl reaction mixture was mixed with 1 μl 10 mg/ml α-cyano-4-hydroxycinnamic acid matrix and spotted onto the sample plate. The molecular mass of histone peptides was determined on the TOF/TOF 5800 system (AB Sciex, USA) operated at the reflectron mode. Final spectra were obtained by calculating the average of 200 shots per position at 200 different positions chosen randomly at each spot. To estimate the pseudo-first-order rate constants, we fit the decrease in the relative intensity of the unmodified peptide over time using a model for a single irreversible reaction [Lys4]_*t*_ = [Lys4]_0_e^−*kt*^, in which [Lys4]_0_ is the initial concentration of the unmodified peptide, [Lys4]_*t*_ represents the concentrations of the unmodified peptide at time *t*, and *k* is the pseudo-first-rate constant. The assays were performed in triplicate, and the averaged data points were used to calculate pseudo-first-order rate constants and fitting errors by using Origin 7 (OriginLab, USA) software.

### Western-blot-based methyltransferase assay

The MLL1 complex was prepared by mixing MLL1_3754–3969_, WDR5_23–334_, ASH2L_FL_ and RBBP5 at the molar ratio of 1:1:1:1 and incubate on ice for 30 minutes without further purification. The reaction assays were performed with 20 μM SAM, 1 μM nucleosome and 1 μM MLL1 complex in the buffer containing 20 mM HEPES, pH 7.8, 10 mM NaCl and 5 mM DTT. The reaction was incubated at 25°C for 1 h. Each reaction mixture was divided into three parts that were separated on a 15% SDS-PAGE. The methylation products of H3K4 were detected by western blot using corresponding antibodies (H3K4me1 antibody, #39297, Active motif; H3K4me2 antibody, # 07-030 Millipore; H3K4me3 antibody, #9751, CST).

### MTase-Glo^TM^ methyltransferase assay

To monitor the progression of the nucleosome-methylation reaction, we used the MTase-Glo™ Methyltransferases Assay Kit (Promega, USA). MLL1 complex (50 nM containing MLL1_3754–3969_, WDR5_23–334_, ASH2L_FL_ and RBBP5 at the molar ratio of 1:1:1:1) and nucleosome (1 μM) were mixed in the buffer of 25 mM HEPES-Na, pH 7.4, 100 mM NaCl, 0.1 mg/ml BSA and 1 mM DTT. The reaction was initiated by adding 20 μM SAM and incubated at 30°C. At different time points (1, 3, 8, 15 min), 8 μl reaction mixture was taken out and stopped by adding 2 μl 0.5% trifluoroacetic acid. Then we followed the protocol described in the kit manual to measure the amount of *S*-adenosyl-l-homocysteine (SAH) produced. The assays were performed in triplicate, and the averaged data points were used for linear fitting of the amount of SAH generated to give an estimation of the initial reaction rate (nM SAH/min).

### Preparation of nucleosome

*Xenopus laevis* histones were expressed in *Escherichia coli* BL21 (DE3) and purified from inclusion bodies as described before (31). The nucleosome was reconstituted from histone octamers and the 167-bp Widom 601 DNA (31). The reconstituted NCP was further purified through Superose 6 increase 10/300 gel filtration.

### Electrophoretic mobility shift assay (EMSA)

The MLL1 complexes were obtained by mixing MLL1_3754–3969_, WDR5_23–334_, ASH2L_FL_ and RBBP5 at the molar ratio of 2:1:1:1, followed by the purification on the Superdex 200 increase (10/300 GL) column. MLL1 complexes were diluted into a serial of concentrations (4–0.125 μM) in six reaction tubes with a final volume of 5 μl in the buffer containing 20 mM Tris–HCl, pH 8.0, 150 mM NaCl, and 10% glycerol. Then 5 μl of 0.2 μM nucleosome was added to each tube. After incubation on ice for 1 h, the reaction mixture was separated on 6% native polyacrylamide gels and visualized by staining with ethidium bromide (EB).

## RESULTS

### Both WD40 and CTD of RBBP5 contribute to activity regulation of MLL1 complex

RBBP5_WDRP_ (residues 330–381) is the primary region required for activity stimulation of KMT2-family complexes (15). However, our previous data showed that an RBBP5_WDRP_-ASH2L_286–505_ complex stimulated MLL3 HKMT activity to a level of ∼70% of full-length RBBP5-ASH2L (15). Results from Wilson lab also declared that the HKMT activity of MLL1 complex assembled with RBBP5_325–380_ was ∼60% of that with RBBP5_1–380_ (17). These studies suggest that other regions of RBBP5, including the WD40 propeller and the C-terminal distal region (Figure 1A), may contribute to fine-tuning the activity of the KMT2-family complexes. Thus, we first quantitatively characterized the HKMT activities of the MLL1 core complexes in the presence of different RBBP5 domains by a mass-spectrometry-based methyltransferase assay. We used an H3 peptide as the substrate. As expected, WDR5_23–334_–ASH2L_FL_–RBBP5_330–381_ substantially stimulated the HKMT activity of MLL1_WIN-SET_ (*k* = 0.23 ± 0.02 h^−1^) (Figure 1B and Supplementary Figure S1), confirming the previous notion that RBBP5_WDRP_ is the major region required for activation of the MLL1 complex (15). We found that the activity of the MLL1 complex containing full-length RBBP5 (*k* = 0.82 ± 0.04 h^−1^) was ∼3.5-fold higher than that of RBBP5_WDRP_-containing complex, indicating that either the WD40 propeller or the CTD region of RBBP5 may also participate in the activation of the MLL1 complex. However, the inclusion of either WD40 (RBBP5_2–381_) or CTD (RBBP5_330–538_) only slightly changed the activity, and the activity boost was only observed in RBBP5_2–538_ containing both WD40 and CTD (Figure 1B). These results imply that the WD40 propeller and the CTD region of RBBP5 have a synergistic effect on stimulating the methyltransferase activity of the MLL1 complex.

One possible explanation for the activity-stimulating effect of the WD40 propeller and the CTD of RBBP5 is that these two regions may cooperatively facilitate MLL1 complex assembly. Indeed, GST pull-down assays showed that GST-ASH2L_FL_–RBBP5_2–381_ pulled down more WDR5 and MLL1 than GST-ASH2L_FL_–RBBP5_330–381_ did (Figure 1C), indicating WD40 propeller of RBBP5 enhanced the stability of the MLL1 complex. RBBP5_2–538_ that includes the CTD region further increased the binding amount of WDR5 and MLL1 to GST-ASH2L_FL_ (Figure 1C). Therefore, we hypothesized that the WD40 and CTD regions of RBBP5 might provide additional binding interfaces for other proteins in the MLL1 core complex to facilitate its assembly.

### The internal interaction between RBBP5_WD40_ and RBBP5_CTD_

**Figure 2.**
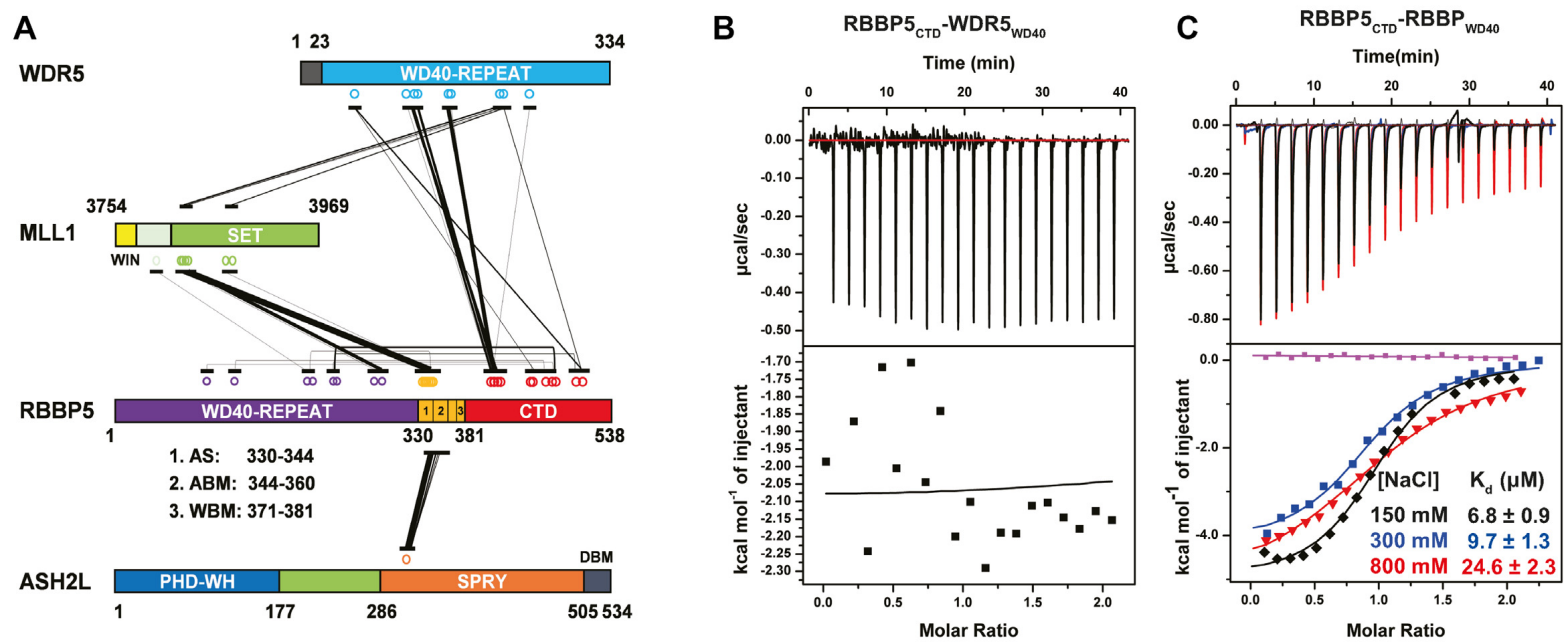
The internal interactions in RBBP5. (**A**) Schematic representation of inter-protein crosslinks detected by CX-MS analysis of the MLL1 complex. RBBP5 intra-protein crosslinks are also labeled. The width of the linking line is correlated to the peptide amount of one specific crosslink appeared in the MS. (**B**) WDR5_23–334_ has no interaction with RBBP5_CTD_ (residues 381–538) as shown by a representative ITC binding curve. The assay buffer is 150 mM NaCl, 25 mM Tris–HCl, pH8.0. (**C**) RBBP5_WD40_ (residues 2–333) has a direct interaction with RBBP5_CTD_ (residues 381–538) as shown by representative ITC binding curves at three different salt concentrations (150, 300 and 800 mM NaCl in the presence of 25 mM Tris–HCl, pH 8.0 buffer). The binding affinity slightly decreased with the increase of salt concentration. The purple curve is the buffer titrated with RBBP5_CTD_. The dissociation constants (*K*_d_) and the reported fitting errors were determined from the representative ITC curves by data fitting using one-site binding model.

Then we employed crosslinking mass spectrometry (CX-MS) to map potential RBBP5-mediated interaction sites in the MLL1 complex. We purified the MLL1_3754–3969_–WDR5_23–334_–RBBP5_FL_–ASH2L_FL_ complex and subjected it to chemical crosslinking using a zero-length crosslinker, EDC-Sulfo-NHS. The crosslinked products were digested by trypsin and then analyzed by liquid chromatography-mass spectrometry. From the MS data, we identified 37 intermolecular cross-links and 38 intramolecular cross-links (Figure 2A and Supplementary Figure S2). Most of the crosslinked patterns confirmed the previously-known interaction interfaces (Figure 2A). For example, ASH2L_SPRY_ (residues 286–505) is in close contacts with RBBP5 ASH2L-binding motif (ABM, residues 344–360). SET-N and SET-I modules of MLL1_SET_ were crosslinked with RRBP5_AS+ABM_ fragments. Unexpectedly, we observed extensive crosslinking between RBBP5_381–538_ (named as RBBP5_CTD_) and WDR5 WD40 propeller. In addition, RBBP5_CTD_ was also intra-molecularly crosslinked to its own WD40 propeller domain (Figure 2A and Supplementary Figure S2). This result indicates that RBBP5_CTD_ has a physical interaction to these two WD40 propellers or they may be spatially close.

To test whether RBBP5_CTD_ can directly bind these two WD40 propellers in WDR5 and RBBP5, we used isothermal titration calorimetry (ITC) to characterize their interactions. No interaction was detected between RBBP5_CTD_ and WDR5_WD40_ (residues 23–334) in our assay condition (Figure 2B), suggesting that they may be in spatial proximity. In contrast, RBBP5_CTD_ interacted with RBBP5 WD40 propeller (residues 2–333) with a dissociation constant (*K*_d_) of 6.8 ± 0.9 μM at 150 mM NaCl concentration (Figure 2C). The binding of RBBP5_CTD_ to its WD40 propeller was primarily driven by hydrophobic interactions because increasing salt concentrations only mildly reduced the affinity between these two domains (Figure 2C).

### RBBP5_CTDM_ wraps around RBBP5_WD40_

**Figure 3.**
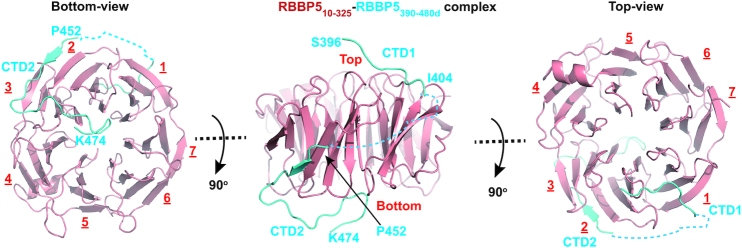
Crystal structure of the RBBP5_WD40_-RBBP5_CTDM_ complex. Three views of the RBBP5_WD40_–RBBP5_CTDM_ complex are shown. RBBP5_WD40_ is colored in red and RBBP5_CTDM_ in cyan. The two segments of RBBP5_CTDM_, CTD1 and CTD2, are indicated. The surface where CTD1 binds is defined as the top of the WD40 propeller.

To further characterize the internal interaction between WD40 propeller and C-terminal regions of RBBP5, we generated a series of RBBP5 C-terminal constructs to assess their interactions with RBBP5_2–333_ by ITC assays (Supplementary Figure S3A and B). A minimal RBBP5 C-terminal construct (390–480) retained the ability to bind with RBBP5_2–333_ (Supplementary Figure S3A and B). After extensive screening, we found that deletion of a disordered loop (residues 422–443) from RBBP5_390–480_ (RBBP5_390–480d_) promoted the crystallization of the complex composed of RBBP5_390–480d_ and RBBP5_10–325_ (Supplementary Figure S3A and B). Hereafter, we refer to RBBP5_390–480d_ as RBBP5_CTDM_ (C-Terminal Distal Minimized) and RBBP5_10–325_ as RBBP5_WD40_. Selenomethionine-substituted crystals diffracted up to 1.8 Å resolution, and we were able to solve the complex structure by single-wavelength anomalous dispersion (Table 1). The electron density map unambiguously allowed us to build the atomic model of the majority of RBBP5_WD40_ (residues 12–324) and two segments of RBBP5_CTDM_ (residues 396–404 and 452–474) (Figure 3 and Supplementary Figure S4A-C).

**Table 1.** Data collection, phasing and refinement statistics for the crystal structure

	SeMet RBBP5_10–325_-RBBP5_390–480d_
**Data collection**	
Space group	*P*1
Cell dimensions	
*a, b, c* (Å)	57.6, 68.8, 72.8
α, β, γ (°)	99.2, 99.3, 113.8
Wavelength (Å)	0.9785(Se peak)
Resolution (Å)	50–1.8
*R*_merge_	0.08(0.42) *
*I*/σ*I*	20.0 (2.5)
Completeness (%)	96.3 (89.2)
Redundancy	6.7/5.5
**Refinement**	
Resolution (Å)	34.8–1.8
No. reflections	86 883
*R*_work_ */R*_free_ (%)^b^	16.5/19.4
No. atoms	
Protein	5387
Solvent & ligands	576
*B*-factors (Å^2^)	
Protein	30.8
Solvent & ligands	45.4
R.m.s. deviations	
Bond lengths (Å)	0.008
Bond angles (°)	0.962

* Highest resolution shell is shown in parenthesis.

RBBP5_WD40_ forms a canonical β-propeller fold with seven WD40 repeats (Figure 3A). The seventh repeat is comprised of three strands from the C-terminal region (RBBP5_296–324_) and one strand from the N-terminal region (RBBP5_12–22_), thereby sealing the β-propeller. The RBBP5_WD40_ conformation is almost identical to that of apo-RBBP5_WD40_ with a root-main-square deviation value of 0.39 Å for 319 C_α_ pairs, indicating that CTDM binding induces no dramatic structural rearrangement of RBBP5_WD40_ (Supplementary Figure S4D). Some loop regions of WD40 propeller contacting CTDM slightly change their configurations (Supplementary Figure S4D).

RBBP5_CTDM_ exhibits an extended conformation and wraps the WD40 propeller through two segments, burying 1472 Å^2^ of the surface area at the interface. The first segment of RBBP5_CTDM_ (CTD1) contains residues 396–404 and sits on the top surface of RBBP5_WD40_. The second segment (CTD2) meanders its way around the side face of RBBP5_WD40_ and also pairs with the repeat 2 of the WD40 propeller to form a five-stranded β-sheet. After a sharp turn, CTD2 further extends along the bottom face and ends in the central cavity of the β-propeller fold (Figure 3). CTD1 and CTD2 are connected by a disordered loop which is invisible in the crystal structure.

### The interfaces between RBBP5_CTD1_ and RBBP5_WD40_

**Figure 4.**
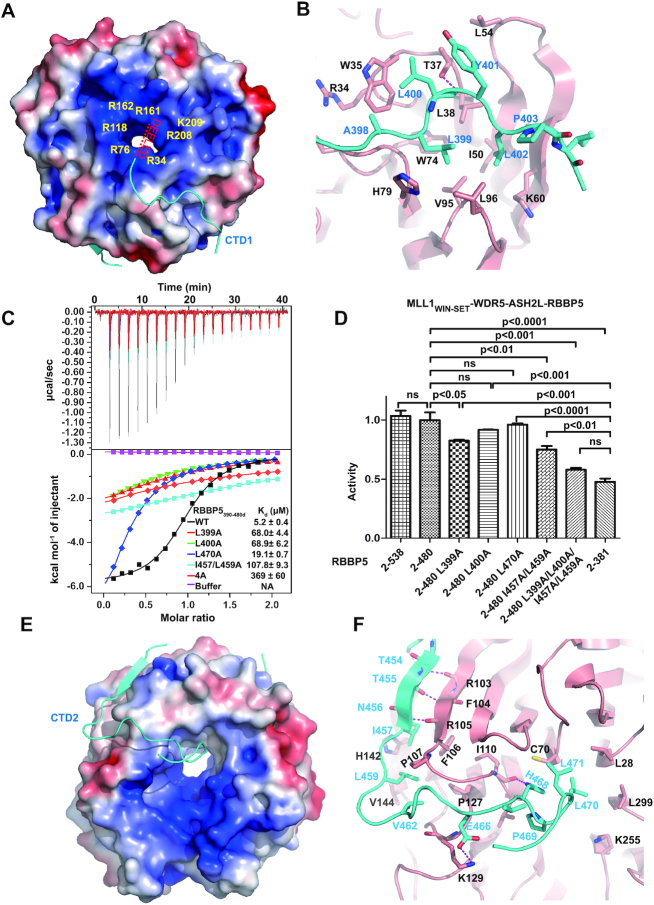
The interaction interface between RBBP5_WD40_ and RBBP5_CTDM_. (**A**) The RBBP5_CTD1_-binding pocket on RBBP5_WD40_. RBBP5_WD40_ surface is colored according to its electrostatic potential (positive potential, blue; negative potential, red). RBBP5_CTD1_ is shown in cyan. One possible path of the absent N-terminal acidic extension of RBBP5_CTD1_ is indicated. (**B**) Details of hydrophobic interactions between RBBP5_WD40_ and RBBP5_CTD1_. The critical residues are presented as ball-and-stick models. RBBP5_WD40_ residues are colored in red and RBBP5_CTD1_ residues in cyan. Hydrogen bonding interactions are shown as dashed magenta lines. (**C**) Effects of mutations in the RBBP5_CTDM_ on the interaction between RBBP5_2–333_ and RBBP5_CTDM_ (RBBP5_390–480d_) analyzed by ITC assays. ITC buffer is 25mM Tris–HCl, 300 mM NaCl, pH 8.0. The RBBP5–4A mutation is L399A/L400A/I457A/L459A mutation. The dissociation constants (*K*_d_) and the reported fitting errors for each mutant were determined from one representative ITC curve by data fitting using one-site binding model. (**D**) HKMT activities of the MLL1 complexes (MLL1_3754–3969_–WDR5_23–334_–ASH2_FL_–RBBP5) containing different RBBP5 mutants determined by the MALDI-TOF-based methyltransferase assays. The HKMT activities are normalized to the activity of RBBP5_2–480_-containing MLL1 complex. Mean ± s.d. (*n* = 3) are shown. The ANOVA test was used to determine the statistical difference of HKMT activities between groups. The label ‘ns’ stands for ‘not significant’ (*P*> 0.05). (**E**) The RBBP5_CTD2_-binding surface on RBBP5_WD40_. RBBP5_WD40_ surface is colored according to its electrostatic potential (positive potential, blue; negative potential, red). RBBP5_CTD2_ is shown in cyan. (**F**) Details of hydrophobic interactions between RBBP5_WD40_ and RBBP5_CTD2_. The critical hydrophobic residues are presented as ball-and-stick models. Salt bridge and hydrogen bonding interactions are shown as dashed magenta lines.

The first segment of CTDM (CTD1) is rich in non-polar residues (_396_SKALLYLPI_404_) and runs across a hydrophobic groove formed by repeats 1 and 2 of RBBP5_WD40_ (Figures 3 and 4A). Three leucine residues (L399, L400 and L402) in CTD1 fit snugly into the hydrophobic groove formed by residues from RBBP5_WD40_, including W35, L38, I50, W74, V95, L96, and the aliphatic side chain of K60 (Figure 4B). Consistent with the structural model, alanine substitution of hydrophobic residues on RBBP5_CTDM_ (L399A, L400A) resulted in ∼13-fold decreased binding affinities between between RBBP5_2–333_ and RBBP5_CTDM_ (Figure 4C). The activity assays showed that RBBP5_2–480_^L399A^ mutation mildly decreased the HKMT activities of MLL1 core complexes and the activity with RBBP5_2–480_^L400A^ mutation was not significantly different from wild type RBBP5_2–480_ (Figure 4D). It suggests that the decreased CTDM-WD40 interactions of RBBP5 L399A and L400A mutations (*K*_d_ ∼ 70 μM) are still strong enough to maintain the activities of MLL1 core complexes.

In addition to the hydrophobic contacts observed in the structure, electrostatic interactions also participate in stabilizing RBBP5_WD40_–RBBP5_CTD1_ interaction. Although the acidic N-terminus of RBBP5_CTD1_ (390DEELED395) is invisible in the crystal structure, this N-terminal extension is presumably located near the central arginine ring of the WD40 propeller and may interact with RBBP5_WD40_ through electrostatic attractions (Figure 4A). This model is partly supported by the observation that the RBBP5_393–480_ construct with fewer acidic residues in CTD1 possessed a reduced affinity with RBBP5_2–333_ (Supplementary Figure S3A). In summary, the hydrophobic contacts and accompanying electrostatic interactions determine the specific recognition of RBBP5_CTD1_ by RBBP5_WD40_.

It is worth noting that a similar CTD1 motif in yeast Swd1 also mediates an internal interaction with the Swd1 WD40 propeller (14). Superimposition of the WD40 domains from hRBBP5 and *Kl*Swd1 reveals that a portion of Swd1_CTD1_ adopts a similar configuration as RBBP5_CTD1_ and binds to a hydrophobic cleft of Swd1_WD40_ (Supplementary Figure S5A-D). Notably, RBBP5_CTD1_ from higher organisms contains a hydrophobic core flanked by two acidic segments, but yeast orthologs have much fewer acidic residues (Supplementary Figure S5B). Correspondingly, the RBBP5 orthologs in yeast species also do not contain the arginine-ring residues that are involved in CTD1-binding (Supplementary Figure S5A), so the acidic feature of Swd1_CTD1_ may not be necessary for its association with the WD40 propeller. Another major difference between human RBBP5_CTD1_ and yeast Swd1_CTD_ is that yeast CTD1 ends with a short α helix binding to the WDR5 ortholog, Swd3 (Supplementary Figure S5C). Swd1_CTD1_ is absolutely essential for binding Swd3 and removal of this short CTD1 completely abolished Swd3-Swd1 interaction (14). On the contrary, hRBBP5_CTD1_ only plays a negligible role in stabilizing WDR5–RBBP5 interaction, because full-length RBBP5 (RBBP5_2–538_) and RBBP5_WDRP_ (RBBP5_330–381_) have comparable binding affinities with WDR5 (Supplementary Figure S5E). Taken together, the CTD1 motif of RBBP5 is a conserved module and has gained structural and functional plasticity in the process of evolution.

### The interface between RBBP5_CTD2_ and RBBP5_WD40_

The second segment of RBBP5_CTDM_ wraps the side and the bottom faces of RBBP5_WD40_. The absence of CTD2 in yeast species (Supplementary Figure S5B) indicates a more specific role of CTD2 in higher organisms. The CTD2 motif is docked to a relatively hydrophobic pocket formed by repeats 2, 3 and 4 (Figures 3 and 4E). The interface between RBBP5_CTD2_ and RBBP5_WD40_ consists of a set of hydrophobic residues, including I457, L459, V462, P469, L470 and L471 from RBBP5_CTD2_ and L28, C70, F106, I110, L111, P127, V133, V144 and L299 from RBBP5_WD40_ (Figure 4F). Mutations of the hydrophobic residues (L470A, I457A and L459A) on CTD2 diminished the interaction between RBBP5_CTDM_ and RBBP5_2–333_ (Figure 4C). Combined mutation of CTD1 and CTD2 (L399A/L400A/I457A/L459A, named as RBBP5_CTDM_-4A mutant) further disrupted the RBBP5_CTDM_–RBBP5_2–333_ interaction (Figure 4C) and reduced the HKMT activity of the MLL1 complex to the activity level comparable with that of the complete-CTD-deletion construct (RBBP5_2–381_) (Figure 4D). These results reinforced the notion that both CTD1 and CTD2 are required for stable association with RBBP5_WD40_, and this internal interaction fine-tunes the methyltransferase activity of the MLL1 complex.

### The internal interaction of RBBP5 compacts the MLL1 complex

**Figure 5.**
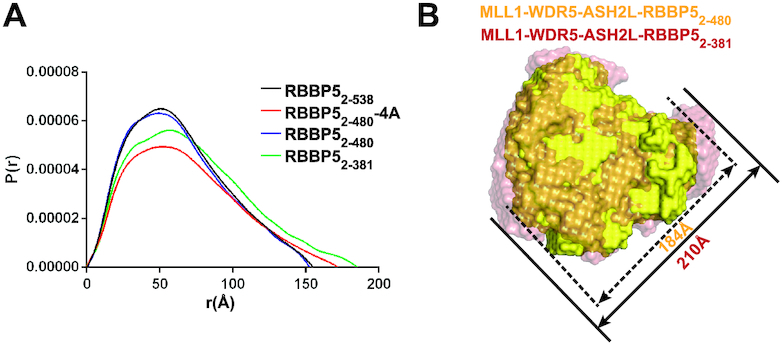
SAXS analyses of MLL1 complex. (**A**) Overlay of *P*(*r*) distributions from the MLL1 complexes assembled from different RBBP5 constructs. RBBP5_2–480_-4A is RBBP5_2–480_ with the L399A/L400A/I457A/L459A mutation. (**B**) Superimposition of the molecular envelops of the RBBP5_2–480_-containing MLL1 complex (yellow) and the RBBP5_2–381_-containing MLL1 complex (red). The sizes of the longest dimension of different complexes are indicated.

To further investigate how the internal interaction of RBBP5 contributes to the assembly of the MLL1 core complex, we utilized small-angle X-ray scattering (SAXS) to characterize the conformation of the MLL1_3754–3969_–WDR5_23–334_–ASH2L_FL_–RBBP5 complexes in the presence of different RBBP5 fragments. The Guinier regions of the scattering curves are linear at low *q* (range of momentum transfer), indicating that samples are non-aggregated (Supplementary Figure S6A). The SAXS data were used to calculate the maximum particle dimension (*D*_max_) and the radius of gyration (*R*_g_). The complexes with RBBP5_2–480_ and RBBP5_2–538_ have similar *D*_max_ and *R*_g_ values (Figure 5A and Supplementary Table S1). In contrast, the MLL1 complex assembled with RBBP5_2–381_ had much larger *D*_max_ and *R*_g_ values, indicating that deletion of RBBP5 CTD (residues 381–538) resulted in an extended conformation of the MLL1 complex (Figure 5A and Supplementary Table S1).

The SAXS data were used to calculate the *ab initio* model for each complex (Supplementary Figure S6B). Rigid body superposition clearly showed that the molecular envelope of the RBBP5_2–480_–containing MLL1 complex is similar to the RBBP5_2–381_-containing complex, but the longest dimension of the RBBP5_2–480_–containing MLL1 complex (184 Å) expands to 210 Å in the RBBP5_2–381_-containing complex (Figure 5B). This result indicates that the existence of CTD compacts the overall conformation of the MLL1 complex. In keeping with this idea, the 4A mutant of RBBP5_2–480_, which disrupted RBBP5_CTDM_–RBBP5_WD40_ interaction (Figure 4C), generated an extended MLL1 complex with increased *D*_max_ and *R*_g_ values (Figure 5A and Supplementary Table S1). These SAXS analyses steadily suggest that the RBBP5_WD40_–RBBP5_CTD_ interaction plays a vital role in promoting the MLL1 complex to maintain a compact conformation required for efficient methylation reactions.

### A novel CTD3 motif is important for methylation on nucleosomes

**Figure 6.**
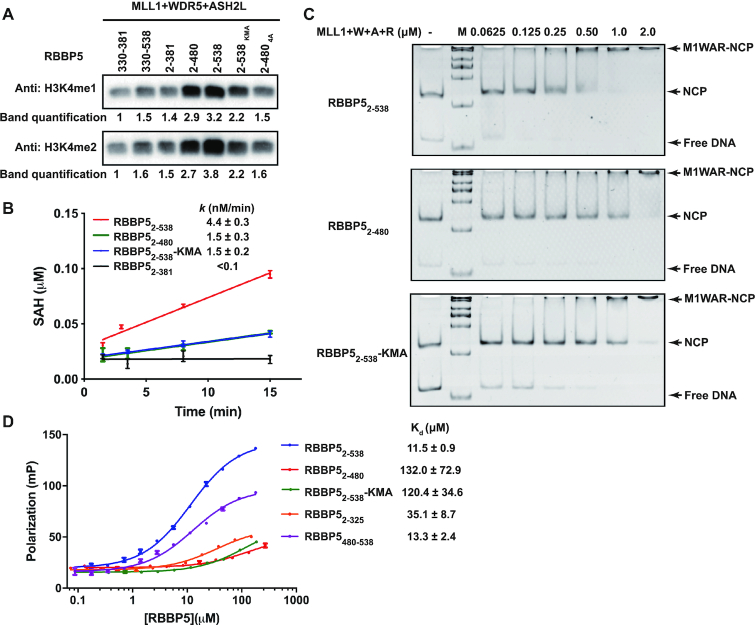
RBBP5_CTD3_ contributes to methylation on nucleosomes. (**A**) Methyltransferase activity assays were carried out using 167-bp nucleosomes as substrates. MLL1 complex (1 μM), nucleosome (1 μM), and SAM (20 μM) were incubated at 25°C for 1 h. At this condition, no H3K4me3 signal was detected. The band densities were quantified by setting the band density from the sample of MLL1_3754–3969_–WDR5_23–334_–ASH2L_FL_–RBBP5_330–381_ as one. RBBP5 2–538_KMA_ is a mutation with 10 lysine residues in CTD3 mutated into alanine. RBBP5 2–480_4A_ contains the RBBP5_2–480_ L399A/L400A/I457A/L459A mutation. (**B**) Time-course monitoring of methyltransferase activities of different MLL1 complexes by MTase-Glo™ Methyltransferases Assay Kit. The assay system contains 50 nM MLL1 complex, 1 μM nuclesome, and 20 μM SAM. Each data point was presented as mean ± s.d. from triplicate measurements. The reaction rate and reported fitting errors were derived by linear fitting of the averaged dataset. (**C**) EMSA showed that MLL1 complexes bound to 167-bp nucleosomes. 0.1 μM nucleosome was incubated with increasing amounts of MLL1 complexes with different RBBP5 constructs as indicated. (**D**) The dissociation constants of the RBBP5-DNA interactions were determined by fluorescence polarization assays. 0.1 μM FAM-labelled 167-bp dsDNA was used. Each data point was shown as mean ± s.d. from triplicate measurements. Some error bars were not shown because the error bar was shorter than the size of the symbol. The dissociation constants (*K*_d_) and the reported fitting errors were determined from the averaged data points by fitting with the sigmoidal dose-response model.

The above studies of MLL1 activity regulation by RBBP5 are limited to the methylation of H3 peptides. Then we are eager to extend the landscape to know if the RBBP5_WD40_-RBBP5_CTD_ interaction is also crucial for histone methylation on nucleosomes. We first tested the HKMT activities of the MLL1 complexes with recombinant mononucleosomes by a western-blot-based methyltransferase assay. The MLL1 complex assembled with RBBP5_WDRP_ (RBBP5_330–381_) has a weak methyltransferase activity on nucleosomes (Figure 6A, lane 1). The inclusion of either the WD40 propeller or CTD only slightly increased the activity of the MLL1 complex (Figure 6A, lanes 2 and 3). A considerable increase in the monomethylation and dimethylation levels of nucleosomal H3 was observed in the MLL1 complex assembled with RBBP5_2–480_, which retains the interaction between RBBP5_WD40_ and RBBP5_CTDM_ (Figure 6A, lane 4). The MLL1 complex assembled with the RBBP5_4A_ mutant that disrupted RBBP5_WD40_-RBBP5_CTDM_ interaction had much weaker methyltransferase activity than the complex containing RBBP5_2–480_ (Figure 6A, lane 7). These results thus confirmed the critical role of the RBBP5_WD40_–RBBP5_CTDM_ interaction in stimulating the methyltransferase activity of MLL1. Interestingly, the inclusion of a C-terminal tail (RBBP5_480–538_) moderately increased the methylation of nucleosomal H3 (Figure 6A, lane 5), indicating an important role of the uncharacterized tail of RBBP5 in activity regulation of the MLL1 complex.

In the western-blot-based methyltransferase assay, equal concentrations of enzymes and the nucleosome substrates were used. The result may not quantitatively describe the activities of the MLL1 complexes containing different RBBP5 fragments since western blot signals can be easily saturated. To accurately compare the activities of the MLL1 complexes, we performed steady-state kinetic analysis of the methyltransfer reaction using 50 nM enzymes and 1 μM substrates. We found that the MLL1 complex assembled with RBBP5_2–381_ had negligible activity towards nucleosomes at such a condition, and the initial reaction rate could not be accurately measured (Figure 6B). Time-course monitoring of methylation progression manifested that RBBP5_2–480_ substantially increased the activity of MLL1 complex on nucleosomes and RBBP5_2–538_ generated a ∼3-fold higher activity than RBBP5_2–480_ (Figure 6B). It is noteworthy that RBBP5_2–538_ and RBBP5_2–480_ had the same methylation activities on H3 peptides (Figure 4D). These data suggest that RBBP5_480–538_ played an additional supporting role in mediating efficient methylation of nucleosomes. Here we name the RBBP5_480–538_ segment RBBP5_CTD3_.

RBBP5_CTD3_ may stimulate the HKMT activity of the MLL1 complex by facilitating the nucleosome binding to the MLL1 complex. To test this idea, we carried out the electrophoretic mobility shift assay (EMSA) to characterize the interactions between MLL1 complexes and nucleosomes. The EMSA data showed that the MLL1 complex assembled with RBBP5_2–538_ had a much higher binding affinity with nucleosomes than the MLL1 complex assembled with RBBP5_2–480_ (Figure 6C). This result thus implies that RBBP5_CTD3_ contains extra binding interfaces with nucleosomes and increases the MLL1 complex association with nucleosomes.

### The CTD3 motif has a DNA-binding activity

Examination of the RBBP5_CTD3_ sequence provides us some clues on how RBBP5_CTD3_ contributes to nucleosome binding. We noticed that RBBP5_CTD3_ is rich of basic residues. There are 14 K/R out of 59 residues in RBBP5_CTD3_ (residues 480–538). We hypothesized that these K/R residues might be involved in DNA binding, thereby increasing the binding of MLL1 complex to nucleosomes. To test this hypothesis, we checked the binding ability of RBBP5 to a fluorescently labeled 167-bp DNA by fluorescence polarization (FP) assay. As expected, RBBP5_CTD3_ bound strongly to DNA (*K*_d_ = 13.3 ± 2.4 μM), comparable to the full-length RBBP5 (*K*_d_ = 11.5 ± 0.9 μM) (Figure 6D). Removal of CTD3 from RBBP5 dramatically disrupted its binding to DNA (the RBBP5_2–480_ curve, Figure 6D). Additionally, we mutated ten lysine residues in CTD3 (K480/481/482/488/491/493/495/500/502/505) to alanine and found that this full-length RBBP5 mutant (RBBP5_KMA_) substantially attenuated the interaction with DNA (Figure 6D). This DNA-binding-deficient RBBP5 mutant also severely impaired the association of the MLL1 complex with nucleosomes (Figure 6C). As a result, the methylation activity of the MLL1 complex assembled with RBBP5_KMA_ was decreased (Figure 6A and B). Collectively, these data reveal that RBBP5_CTD3_ is a novel DNA-binding motif in RBBP5. This new element can stabilize the MLL1 complex associated with nucleosomes and stimulates the HKMT activity of the MLL1 complex on nucleosome H3.

## DISCUSSION

**Figure 7.**
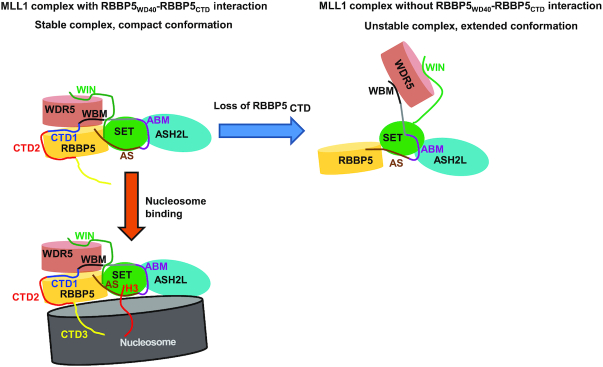
The model for the role of RBBP5_CTD_ in regulating MLL1 complex. The interaction between RBBP5_WD40_ and RBBP5_CTD_ facilitates the formation of a compact MLL1 complex with correct coordination of the structural elements required for methylation. The loss of RBBP5_CTD_ generates an extended complex with low catalytic efficiency. A vertebrate-specific RBBP5_CTD3_ can bind to nucleosome DNA and increases the association of the MLL1 complex with nucleosomes, thereby promoting methylation of nucleosomal H3.

RBBP5 is a conserved scaffold protein to orchestrate the assembly of KMT2-family methyltransferase complexes (13–15). In the present work, we identified an internal interaction in RBBP5 and revealed its roles in MLL1 complex assembly and activity regulation. The dissociation constant for the interaction between RBBP5_WD40_ and RBBP5_CTD_ is around sub micro-molar range as determined by ITC assays (Figure 2C). Given that the binding affinity is measured from two purified proteins separated in solution, the actual interaction between these two regions on a single RBBP5 polypeptide is expected to be much stronger than that reported here. Thus, this internal interaction in RBBP5 might be comparable with other RBBP5-mediated inter-molecular interactions in the MLL1 complex (e.g. WDR5-RBBP5, *K*_d_ = 1.8 ± 0.1 μM; ASH2L-RBBP5, *K*_d_ = 0.46 ± 0.04 μM) (18,32). Such a robust internal interaction may have a profound effect on the assembly of the MLL1 complex. We indeed observed that the interaction between RBBP5_WD40_ and RBBP5_CTD_ helps the MLL1 complex to form a compact conformation, as shown by the SAXS analyses (Figure 5). The compact conformation of the MLL1 complex driven by the RBBP5_WD40_–RBBP5_CTD_ interaction may facilitate the proper coordination of structural elements required for efficient methylation reactions (Figure 7).

Another intriguing finding in our study is the novel DNA-binding CTD3 motif found at the end of RBBP5. It is worth noting that Mittal *et al.* recently reported that RBBP5_WD40_ domain could bind dsDNA (*K*_d_: 29.7 ± 4.2 μM) and ssRNA (*K*_d_: 22.5 ± 3.2 μM), presumably mediated by the arginine-rich surface of WD40 propeller (17). We also found that RBBP5_2–325_ binds to dsDNA with a similar affinity (*K*_d_: 35.1±8.7 μM) (Figure 6D). However, RBBP5_2–480_ that includes WD40, WDRP, CTD1 and CTD2 had a negligible DNA-binding activity (Figure 6D). This result can be explained by our structural model, in which the arginine-rich surface of RBBP5_WD40_ is involved in the binding of the acidic segment of RBBP5_CTD1_ (Figure 4A), thereby masking the potential DNA-binding surface on the WD40 propeller. Instead, the CTD3 motif serves as the primary DNA-binding element in RBBP5, as the mutation in CTD3 substantially decreased the DNA-binding activity of full-length RBBP5 (Figure 6D). This novel DNA-binding element is important for nucleosome recognition, as deletion or mutation of the CTD3 motif decreased the nucleosome-binding affinity of the MLL1 complex (Figure 6C). We propose that when the MLL1 complex binds to nucleosomes, RBBP5 is positioned close to the nucleosome and RBBP5_CTD3_ directly contacts with nucleosomal DNA (Figure 7). RBBP5 CTD3 acts as an additional buckle to secure nucleosome recognition and also helps orient the H3 tail to the active site of MLL1_SET_, thereby increasing the MLL1 methylation activity on nucleosomal substrates. 

Finally, the structural comparison of human RBBP5 and yeast Swd1 allowed us to understand the evolutionary plasticity of RBBP5. The primary sequences of the N-terminal WD40 propeller and the central WDRP region of RBBP5 are well conserved across species (Supplementary Figure S5A). In contrast, the C-terminal distal regions in different species are highly variable (Supplementary Figure S5B). Due to the absence of the CTD2 and CTD3 motifs in RBBP5 yeast orthologs (Supplementary Figure S5B), the internal interaction between WD40 and CTD is not necessarily conserved in yeast species. The CTD2 motif that wraps around RBBP5_WD40_ is specific for animal and plant species. The CTD3 motif involved in nucleosome recognition is exclusively found in animal species. It is worth noting that the differences in CTD3 motifs are closely correlated with the complexity of the animal species. For example, *C. elegans* RBBP5 has a very short CTD3, while vertebrate RBBP5 has a much longer CTD3 with more positively charged residues. Differences in CTD regions strongly support the idea that RBBP5 becomes more complicated during evolution to accommodate its role in precise regulation of gene expression in higher organisms. How RBBP5 mediates functional specification of the KMT2-family methyltransferase complexes in different species awaits further investigation.

## DATA AVAILABILITY

Coordinate and structure factor have been deposited in the Protein Data Bank under accession code 6KM7 (RBBP5_10–325_-RBBP5_390–480d_). The SAXS data have been deposited in the Small Angle Scattering Biological Data Bank with the accession codes: SASDGD4, MLL1 complex with RBBP5_2–381_; SASDGE4, MLL1 complex with RBBP5_2–480_; SASDGF4, MLL1 complex with RBBP5_2–480_-4A mutant; SASDGG4, MLL1 complex with RBBP5_2–538_.

## Supplementary Material

gkz819_Supplemental_File
