# Supplementary material for: The internal interaction in RBBP5 regulates assembly and activity of MLL1 methyltransferase complex
Source: Nucleic Acids Res. 2019 Sep 23;47(19):10426–38. doi: 10.1093/nar/gkz819 (PMC6821195; doi:10.1093/nar/gkz819)
Supplement: gkz819_Supplemental_File [file gkz819_supplemental_file.pdf]

The internal interaction in RBBP5 regulates assembly and activity of MLL1 methyltransferase complex

[1]Jianming Han, [1]Tingting Li, [1]Yanjing Li, [2]Muchun Li, [1]Xiaoman Wang, [3,4]Chao Peng, [3,4]Chen Su, [3]Na Li, [3]Yiwen Li, [1]Ying Xu, [1,2,4]Yong Chen

[1]State Key Laboratory of Molecular Biology, National Center for Protein Science Shanghai, CAS Center for Excellence in Molecular Cell Science, Shanghai Institute of Biochemistry and Cell Biology, Chinese Academy of Sciences; University of Chinese Academy of Sciences, 333 Haike Road, Shanghai 201210, China.

[2]School of Life Science and Technology, Shanghai Tech University, 100 Haike Road, Shanghai 201210, P. R. China

[3]National Facility for Protein Science in Shanghai, Zhangjiang Lab, Shanghai, 201210, China.

[4]Shanghai Science Research Center, Chinese Academy of Sciences, Shanghai, 201204, China.

Correspondence should be addressed to Y.C. (yongchen@sibcb.ac.cn)

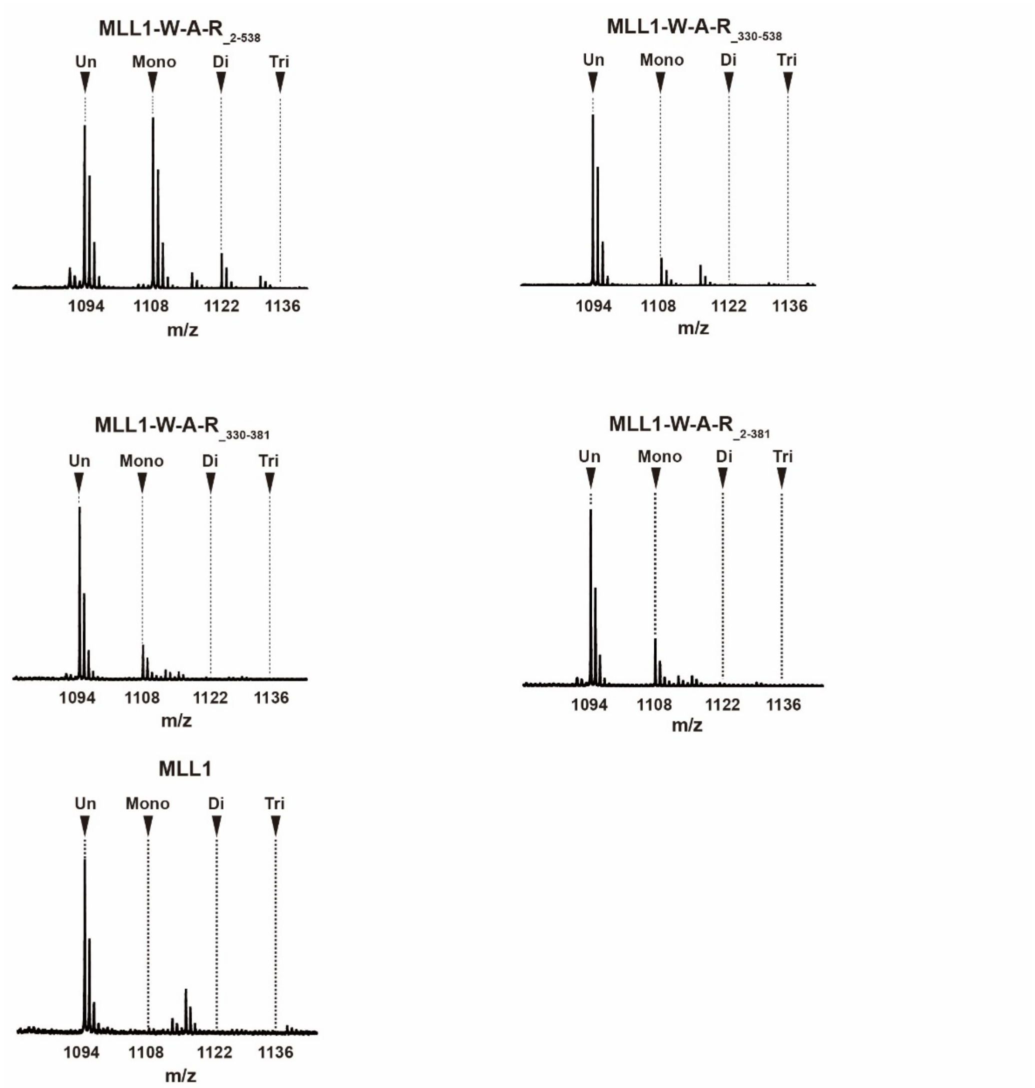

Supplementary Figure 1. Representative MALDI-TOF spectra after 1-hour reaction for MLL1 complexes assembled with different RBBP5 fragments. MLL1 complex with full-length RBBP5 (RBBP5 $_{2\text{-}538}$) is more active than others, as reflected by the higher mono-methylated H3 peak. The peaks for unmodified (Un) and mono-, di-, tri-methylated products are labeled.

A

Protein1(site)-Protein2(site)	#Spec-Total	Best Score
MLL1(3828)-RBBP5(338)	32	8.61E-29
MLL1(3825)-RBBP5(338)	7	1.32E-29
MLL1(3829)-RBBP5(288)	18	1.86E-25
MLL1(3828)-RBBP5(340)	8	6.06E-23
MLL1(3828)-RBBP5(349)	1	1.59E-02
MLL1(3828)-RBBP5(344)	1	5.01E-02
MLL1(3870)-RBBP5(338)	1	8.16E-03
MLL1(3825)-RBBP5(340)	1	3.45E-10
MLL1(3825)-RBBP5(341)	2	6.62E-05
MLL1(3799)-RBBP5(209)	1	6.17E-02
ASH2L(311)-RBBP5(338)	31	6.42E-19
MLL1(3796)-RBBP5(209)	1	6.92E-08
MLL1(3824)-RBBP5(338)	2	3.12E-05
MLL1(3824)-RBBP5(340)	1	6.00E-04
MLL1(3828)-RBBP5(341)	5	2.17E-21
ASH2L(311)-RBBP5(344)	3	3.01E-16
ASH2L(311)-RBBP5(341)	4	3.95E-15
ASH2L(311)-RBBP5(340)	7	2.65E-12
RBBP5(410)-WDR5(120)	16	4.67E-10
RBBP5(412)-WDR5(120)	4	1.99E-08
RBBP5(407)-WDR5(120)	12	8.03E-08
RBBP5(407)-WDR5(112)	1	2.45E-05
RBBP5(407)-WDR5(123)	13	2.61E-07
RBBP5(407)-WDR5(159)	21	4.26E-08
RBBP5(407)-WDR5(162)	1	1.25E-04
RBBP5(407)-WDR5(247)	1	4.12E-03
RBBP5(413)-WDR5(120)	4	1.05E-04
RBBP5(450)-WDR5(58)	2	4.53E-08
MLL1(3828)-RBBP5(280)	1	2.04E-05
MLL1(3878)-WDR5(211)	5	4.85E-08
MLL1(3828)-WDR5(211)	4	4.59E-05
MLL1(3828)-WDR5(212)	1	4.00E-13
RBBP5(238)-WDR5(162)	1	5.18E-02
WDR5(58)-RBBP5(505)	5	3.49E-07
WDR5(211)-MLL1(3825)	3	6.86E-07
WDR5(211)-RBBP5(505)	2	1.09E-03
MLL1(3878)-WDR5(212)	1	7.09E-15

B

Protein1(site)-Protein2(site)	#Spec-Total	Best Score
RBBP5(149)-RBBP5(172)	23	1.31E-29
RBBP5(150)-RBBP5(172)	32	1.43E-23
RBBP5(148)-RBBP5(172)	15	3.34E-13
RBBP5(209)-RBBP5(338)	3	1.10E-27
RBBP5(149)-RBBP5(174)	13	1.11E-20
RBBP5(150)-RBBP5(174)	15	1.42E-20
RBBP5(148)-RBBP5(174)	10	1.80E-17
RBBP5(164)-RBBP5(179)	5	2.10E-17
RBBP5(152)-RBBP5(172)	4	9.47E-14
MLL1(3873)-MLL1(3949)	7	1.22E-11
RBBP5(174)-RBBP5(236)	1	3.94E-08
ASH2L(299)-ASH2L(310)	7	1.02E-07
RBBP5(209)-RBBP5(340)	2	1.86E-07
WDR5(27)-WDR5(313)	2	4.02E-07
RBBP5(236)-RBBP5(474)	1	1.70E-05
MLL1(3796)-MLL1(3933)	2	3.43E-05
WDR5(1)-WDR5(292)	2	3.82E-05
RBBP5(129)-RBBP5(466)	2	7.02E-05
MLL1(3797)-MLL1(3933)	2	1.94E-04
RBBP5(209)-RBBP5(344)	1	2.44E-04
RBBP5(129)-RBBP5(150)	1	1.69E-03
RBBP5(209)-RBBP5(341)	2	2.33E-03
MLL1(3799)-MLL1(3933)	1	7.00E-02
RBBP5(99)-RBBP5(449)	1	7.63E-02
MLL1(3795)-MLL1(3828)	1	8.86E-02
RBBP5(236)-RBBP5(174)	3	2.38E-06
RBBP5(238)-RBBP5(481)	1	5.41E-05
RBBP5(238)-RBBP5(482)	3	1.99E-15
RBBP5(238)-RBBP5(488)	2	2.50E-02
RBBP5(240)-RBBP5(482)	2	2.05E-04
RBBP5(495)-RBBP5(238)	2	2.23E-02
RBBP5(513)-RBBP5(505)	3	1.19E-08
RBBP5(536)-RBBP5(493)	1	8.01E-02
RBBP5(536)-RBBP5(517)	1	3.73E-06
RBBP5(536)-RBBP5(482)	5	9.66E-10
RBBP5(536)-RBBP5(505)	5	1.25E-06
RBBP5(536)-RBBP5(488)	2	1.75E-05
RBBP5(338)-RBBP5(209)	1	2.49E-06

Supplementary Figure 2. Crosslinking mass spectrometry (CX-MS) analysis of the MLL1$_{3754\text{-}3969}$-WDR5$_{23\text{-}334}$-ASH2L$_{FL}$-RBBP5$_{FL}$ (M1WAR) complex. The purified M1WAR complex was crosslinked by EDC (1-ethyl-3-[3-imethylaminopropyl] carbodiimide hydrochloride) coupled with Sulfo-NHS and analyzed by MS. This analysis identified 37 pairs of inter-molecular interaction (A) and 38 pairs of intra-molecular interaction (B) with a pLink2 score less than 0.01.

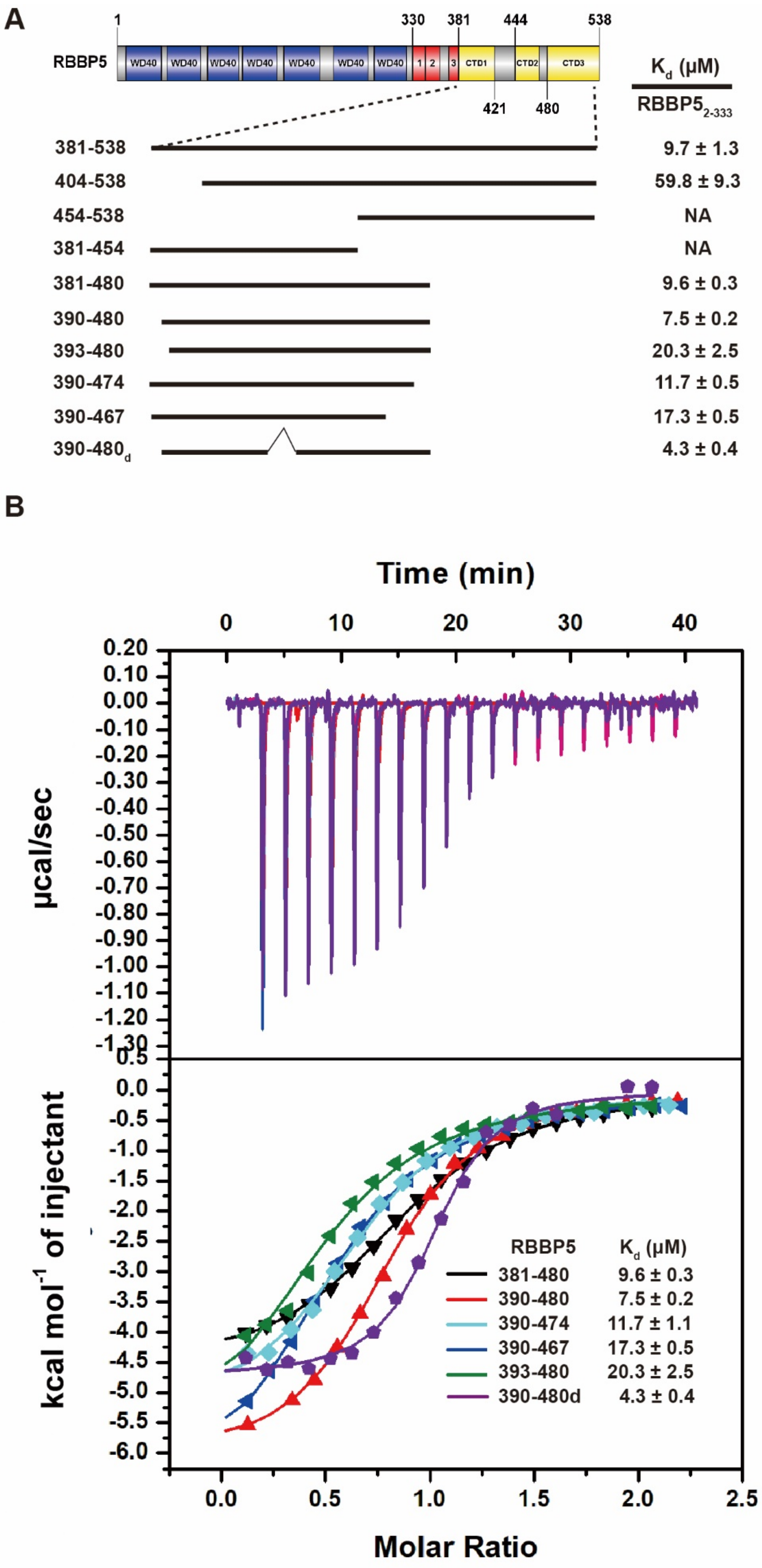

A
RBBP5
1
330
381
444
538
WD40
1
2
3
CTD1
CTD2
CTD3
421
480
K_d (μM)
$RBBP5_{2-333}$
381-538 9.7 ± 1.3
404-538 59.8 ± 9.3
454-538 NA
381-454 NA
381-480 9.6 ± 0.3
390-480 7.5 ± 0.2
393-480 20.3 ± 2.5
390-474 11.7 ± 0.5
390-467 17.3 ± 0.5
$390-480_d$ 4.3 ± 0.4
B
Time (min)
μcal/sec
kcal mol^{-1} of injectant
Molar Ratio
RBBP5 K_d (μM)
381-480 9.6 ± 0.3
390-480 7.5 ± 0.2
390-474 11.7 ± 1.1
390-467 17.3 ± 0.5
393-480 20.3 ± 2.5
390-480d 4.3 ± 0.4

Supplementary Figure 3. Mapping interaction fragments between CTD and WD40 of RBBP5.

A. Schematic summary of different RBBP5 C-terminal constructs tested for binding to $RBBP5_{2-333}$ by isothermal titration calorimetry (ITC) assays. NA stands for no heat change during injections. 390-480d represents $RBBP5_{390-480}$ with a loop deletion from 422 to 443.

B. Representative ITC data for the binding of different RBBP5 C-terminal constructs to $RBBP5_{2-333}$. The upper panel is the heat change upon titration of RBBP5 C-terminal construct into $RBBP5_{2-333}$, and the lower panel is the binding isotherm profile. The assay buffer is 300 mM NaCl, 25 mM Tris-HCl, pH 8.0. The dissociation constants (K_d) and the reported fitting errors were determined from the representative ITC curves by data fitting using one-site binding model.

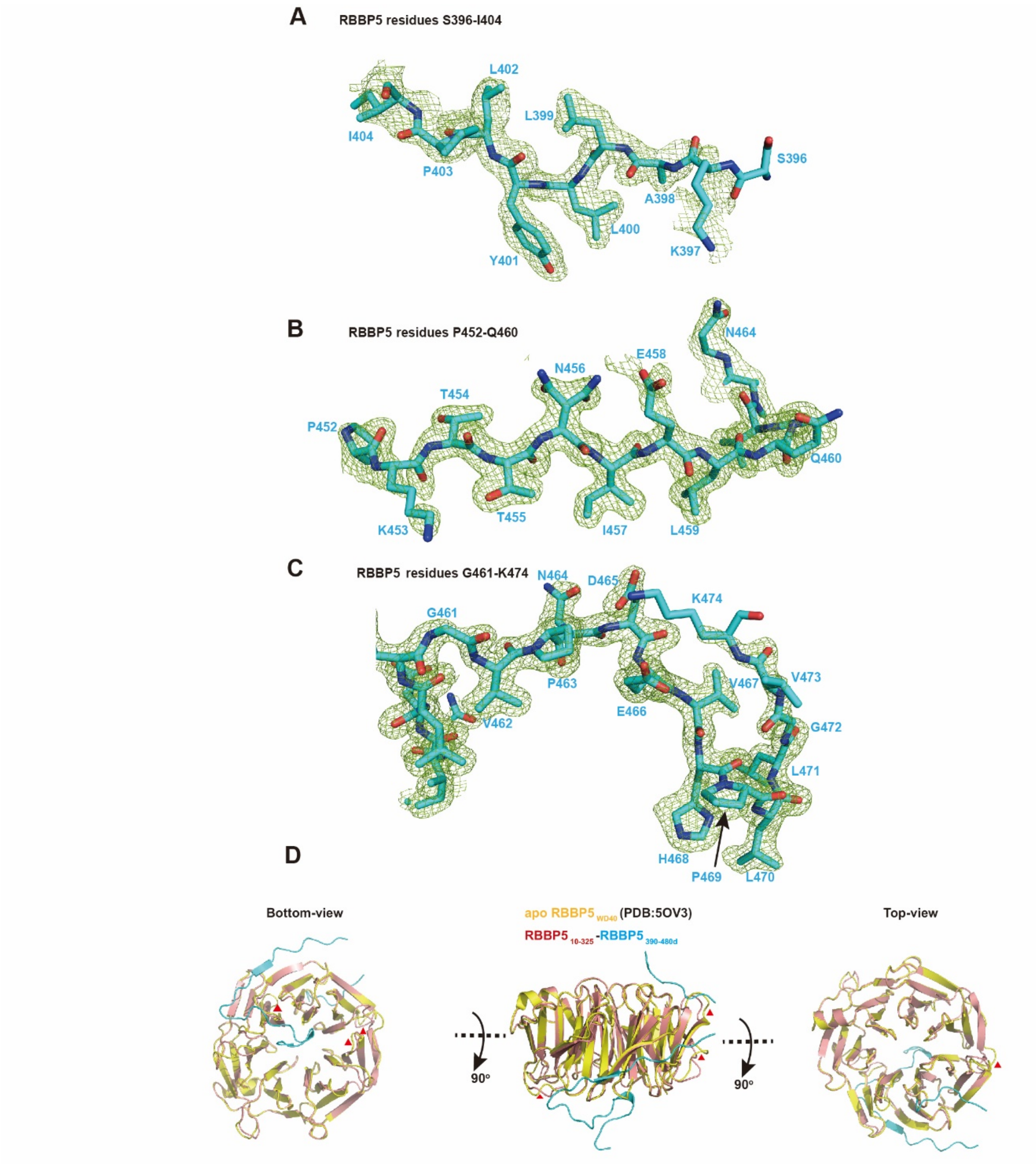

Supplementary Figure 4. The crystal structure of RBBP5$_{WD40}$-RBBP5$_{CTD}$.

A. The Fo-Fc omit map contoured at the 3 δ level is shown for RBBP5$_{CTD1}$ (residues S396-I404).

B. The Fo-Fc omit map contoured at the 3 δ level is shown for the N-terminal part of RBBP5$_{CTD2}$ (residues P452-Q460).

C. The Fo-Fc omit map contoured at the 3 δ level is shown for the C-terminal part of RBBP5$_{CTD2}$ (residues G461-K474).

D. Superimposition of apo WD40 propeller of RBBP5 (PDB: 5OV3) and RBBP5$_{10\text{-}325}$-RBBP5$_{390\text{-}480d}$ complex shows that there is no significant conformational change on WD40 propeller except for some loops contacting RBBP5$_{CTD}$ (labeled with red triangles).

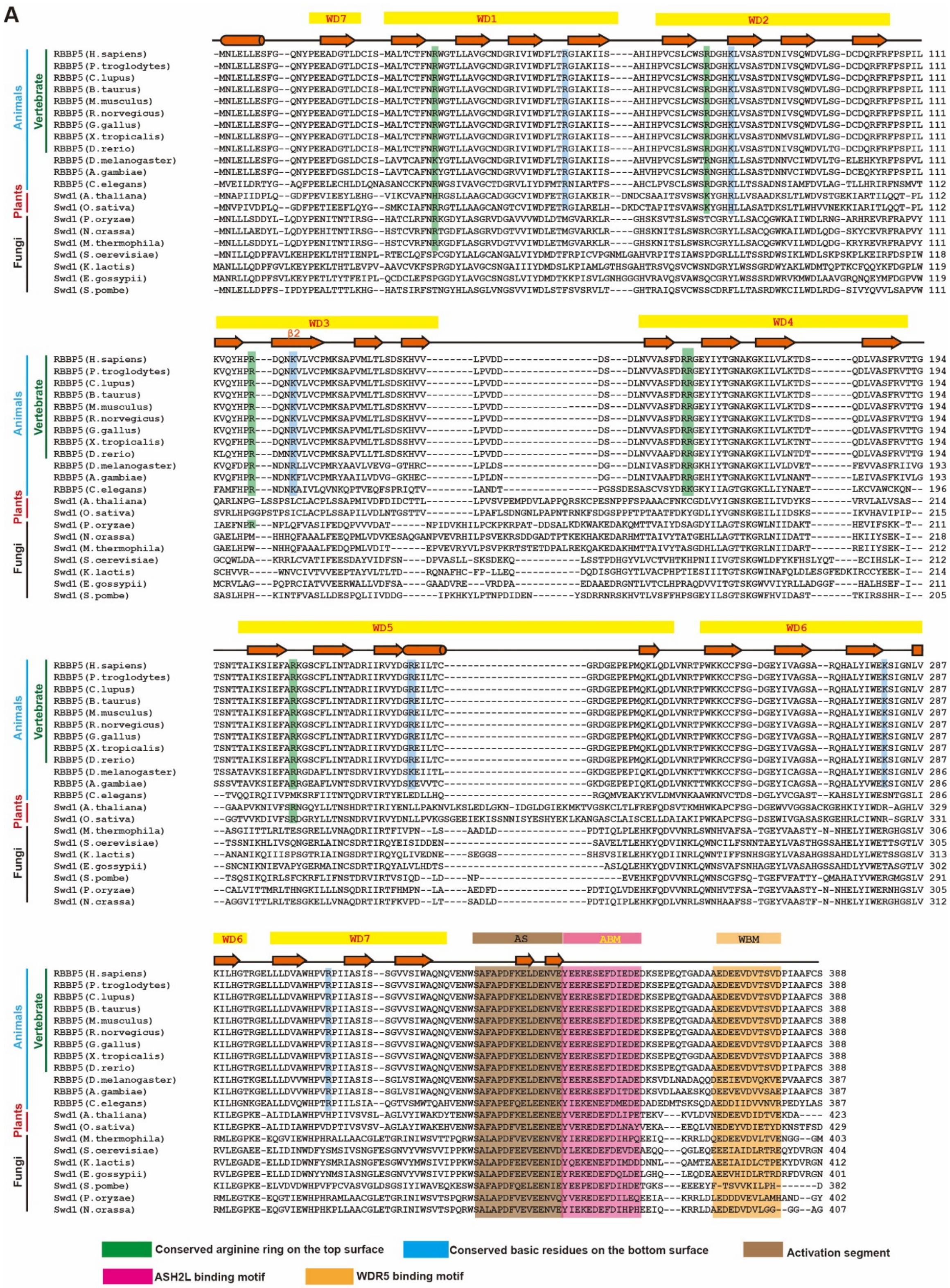

A
WD7
WD1
WD2
WD3
β2
WD4
WD5
WD6
AS
ABM
WBM
Animals
Vertebrate
Plants
Fungi
Conserved arginine ring on the top surface
Conserved basic residues on the bottom surface
Activation segment
ASH2L binding motif
WDR5 binding motif

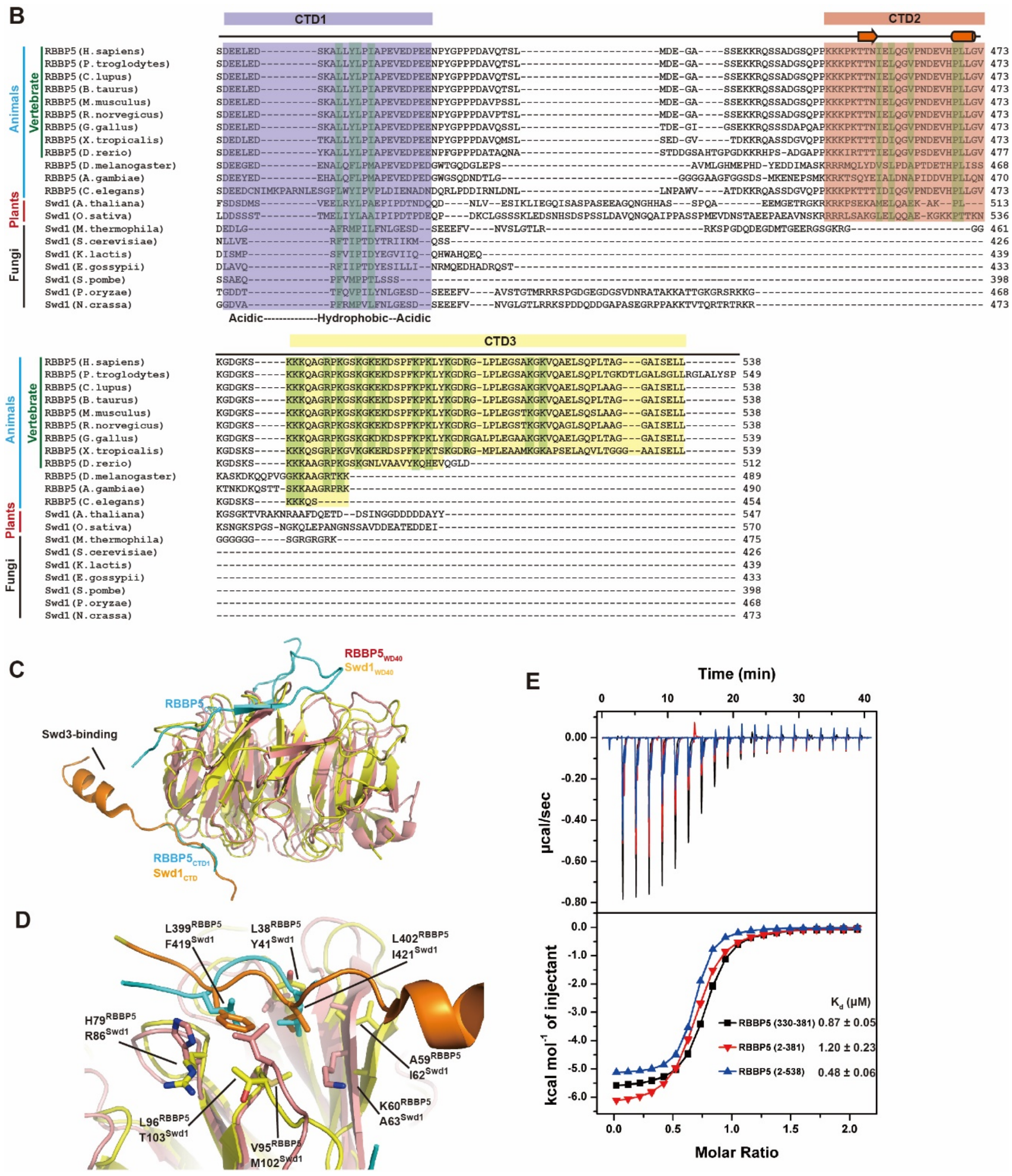

Supplementary Figure 5. Comparison of RBBP5 from different species.

A. Sequence alignment of RBBP5 orthologs from yeast, plant, and animal species. RBBP5 WD40 propeller and WDRP region are shown. Secondary structure assignments based on the hRBBP5 and ySwd1 structures are shown as cylinders (α-helices) and arrows (β strands)

above the sequences. Conserved arginine residues (arginine-ring) at the top surface of WD40 propeller are highlighted in green. The lysine/arginine residues at the bottom of WD40 propeller are highlighted in cyan. These basic residues are conserved in animal species but not in yeast and plant species.

B. Sequence alignment of CTD regions of RBBP5 orthologs from yeast, plant and animal species. The conserved hydrophobic residues in CTD1 and CTD2 are highlighted. The conserved basic residues in CTD3 are also highlighted.

C. Structure superimposition of Swd1 (PDB: 6CHG) and hRBBP5 based on the WD40 propeller. The CTD from Swd1 has an extra helix extension involved in Swd3 binding. $RBBP5_{WD40}$, red; $RBBP5_{CTD1}$, cyan; $Swd1_{WD40}$, yellow; $Swd1_{CTD1}$, orange.

D. The relatively conserved interface between $RBBP5_{WD40}$ and $RBBP5_{CTD1}$. The structural equivalent residues in RBBP5 and Swd1 are indicated.

E. ITC analysis of the RBBP5-WDR5 interaction in the buffer of 150 mM NaCl, 25 mM Tris-HCl, pH8.0. The dissociation constants (K_d) and the reported fitting errors were determined from the representative ITC curves by data fitting using one-site binding model.

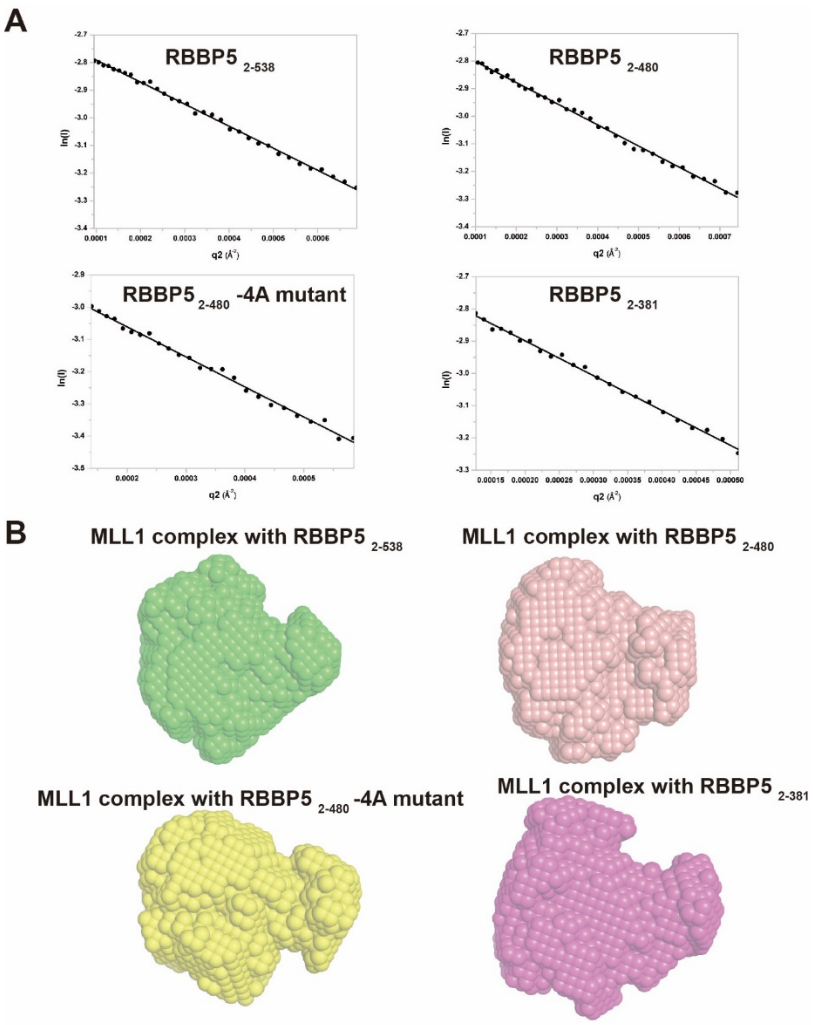

Supplementary Figure 6. Small-angle X-ray scattering analyses of MLL1-WDR5-RBBP5-ASH2L complex.

A. The Guinier plots of the scattering data show a linear fit at low *q*. SAXS data shown were from 2 mg/mL MLL1-WDR5-ASH2L-RBBP5 complexes. MLL1 contains residues 3754-3969, WDR5 contains residues 23-334, and ASH2L is full-length. RBBP5 used are indicated in each panel.

B. The *ab initio* solution structures of MLL1-WDR5-ASH2L-RBBP5 complexes with different RBBP5 constructs.

Supplementary Table 1. Summary of SAXS analyses of $MLL1_{3754-3769}$-$WDR5_{23-334}$-$ASH2L_{FL}$-RBBP5 complexes assembled with different RBBP5 fragments.

(a) Sample details

	2-381	2-480	2-480-4A*	2-538
Organism	*Homo sapiens*			
Source (Catalogue No. or reference)	*E.coli* expressed			
UniProt sequence ID (residues in construct)	Q15291(2-381)	Q15291(2-480)	Q15291(2-480-4A*)	Q15291(2-538)
	Q03164 (3754-3969)	Q03164 (3754-3969)	Q03164 (3754-3969)	Q03164 (3754-3969)
	P61964 (23-334)	P61964 (23-334)	P61964 (23-334)	P61964 (23-334)
	Q9UBL3-3 (1-534)	Q9UBL3-3 (1-534)	Q9UBL3-3 (1-534)	Q9UBL3-3 (1-534)
Extinction coefficient ε (wavelength and units) (A280, M^{-1} cm^{-1})	234130	236690	236690	237970
Molecular mass *M* from chemical composition (KDa)	161.8	172.3	172.1	178.4
Concentration (mg/ml)	0.5-2	0.5-2	0.5-2	0.5-2
Solvent composition and source	300 *mM* NaCl, 25 *mM* Tris pH 8.0, 4% glycerol, 1 *mM* TCEP			

(b) SAS data collection parameters

Source, instrument and description or reference	BL19U2 at SSRF with Pilatus 1M (DECTRIS Ltd)
Wavelength (Å)	0.9184
Beam geometry (size, sample-to-detector distance)	340μm x 60μm (H x V), 2.415m
q-measurement range (Å-1 or nm-1)	0.008-0.47
Absolute scaling method	Comparison with scattering with 1 mm pure H2O
Basis for normalization to constant counts	Take silver behenate as standard to set the mask, then normalize the 2D images
Method for monitoring radiation damage, X-ray dose where relevant	SAXS data were collected as continuous serial exposures and scattering profiles for the passes were compared to monitor the radiation damage
Exposure time, number of exposures	1 s per frame, total 20 frames
Sample temperature (ºC)	10

(c) Software employed for SAS data reduction, analysis and interpretation

SAS data reduction	*ln*(*I*) versus *q2* using OriginPro 8 (http://www.OriginLab.com), solvent substraction using *PRIMUS* (*ATSAS* 2.8.0)
Extinction coefficient estimate	http://protcalc.sourceforge.net/
Basic analyses: Guinier, *P*(r), *V*p	*PRIMUS* (*ATSAS* 2.8.0)
Shape/bead modelling	*DAMMIF* and *DAMAVER*

Molecular graphics	*PyMOL*			

(d) Structural parameters

	2-381	2-480	2-480-4A*	2-538
Guinier Analysis				
I(0) (cm-1)	0.069±0.00032	0.067±0.00023	0.058±0.00021	0.068±0.00018
*R*g (Å)	56.69±1.07	49.83±0.62	53.46±0.82	49.93±0.52
q-range (Å-1)	0.0092-0.0226	0.0097-0.0257	0.0097-0.0241	0.0097-0.0257
Quality-of-fit parameter (fidelity)	0.91	0.79	0.95	0.92
M(KDa) from *I*(0) (ratio to expected value)	171.6 (1.06)	160.8 (0.93)	164.4 (0.96)	193.5 (1.08)
P(r) analysis				
I(0) (cm-1)	0.0682	0.066	0.0574	0.0673
*R*g (Å)	57.07	49.65	53.88	50.16
*d*max (Å)	185.5	152.5	172	155
q-range (Å-1)	0.0092-0.1223	0.0097-0.0.1290	0.0097-0.1316	0.0097-0.1475
Quality-of-fit parameter (Total estimate from *GNOM*)	0.91	0.94	0.93	0.95
M(KDa) from *I*(0) (ratio to expected value)	169.6 (1.05)	158.4 (0.92)	162.7 (0.95)	191.5 (1.07)
Porod volume estimate (Å^3)	360000	256000	313000	282000

(e) Shape modelling results (a complete panel for each method)

q-range for fitting(Å-1)	0.0092-0.3000	0.0097-0.3000	0.0097-0.3000	0.0097-0.3000
Symmetry/anisotropy assumptions	*P*1	*P*1	*P*1	*P*1
Ambiguity measure(s)	Filteriing of averaged *ab initio* models	Filteriing of averaged *ab initio* models	Filteriing of averaged *ab initio* models	Filteriing of averaged *ab initio* models
χ^2 value	1.187	1.36	1.339	1.582
NSD value betten clusters	1.753	1.683	1.778	1.585
Model volume (nm^3)	151.5	167.1	115	159.6
Model precision/resolution (nm)	2.799	2.707	2.833	2.653

(f) Data and model deposition IDs

SASDBD	SASDGD4	SASDGE4	SASDGF4	SASDGG4
（https://www.sasbdb.org/）				

* 4A stands for L399A/L400A/I457A/L459A mutation of RBBP5.
